# SIVEH: Numerical Computing Simulation of Wireless Energy-Harvesting Sensor Nodes

**DOI:** 10.3390/s130911750

**Published:** 2013-09-04

**Authors:** Antonio Sanchez, Sara Blanc, Salvador Climent, Pedro Yuste, Rafael Ors

**Affiliations:** 1 ITACA Institute, Universitat Politècnica de València, Valencia 46022, Spain; E-Mail: scliment@itaca.upv.es; 2 DISCA Department, Universitat Politècnica de València, Valencia 46022, Spain; E-Mails: sablacla@disca.upv.es (S.B.); pyuste@disca.upv.es (P.Y.); rors@disca.upv.es (R.O.)

**Keywords:** energy and resource management, low-power hardware design, numerical computing, wireless sensor networks, energy harvesting, energy neutral operation, simulation model

## Abstract

The paper presents a numerical energy harvesting model for sensor nodes, SIVEH (Simulator I–V for EH), based on I–V hardware tracking. I–V tracking is demonstrated to be more accurate than traditional energy modeling techniques when some of the components present different power dissipation at either different operating voltages or drawn currents. SIVEH numerical computing allows fast simulation of long periods of time—days, weeks, months or years—using real solar radiation curves. Moreover, SIVEH modeling has been enhanced with sleep time rate dynamic adjustment, while seeking energy-neutral operation. This paper presents the model description, a functional verification and a critical comparison with the classic energy approach.

## 1. Introduction

The use of wireless sensor networks (WSNs) is widely extended in monitoring applications. Sensor nodes are usually battery-powered devices with finite energy. However, in many applications, nodes are required to operate for very long periods of time. Energy harvesting (EH) can extend node lifetime indefinitely, although there is a limit on daily power consumption rates. For example, if solar energy is used to recharge batteries or supercapacitors, energy harvested varies along the day and will probably be unavailable at night or in bad weather conditions. Thus, for nodes that operate without penalty on performance during sunny periods, the same power consumption at night could imply a quick loss of energy without being able to recharge.

Energy-aware management policies—which lead to energy-saving operation—are essential to avoid power-off failures on devices or unforeseen disconnection gap time periods with no energy to communicate. Both hardware component consumption and the sensor duty cycle are important factors to determine super- or ultra-capacitor available energy. Although an orthogonal design of the physical layer and power management policies is possible, a cross-layer design has proven to be more efficient, especially on energy constrained systems, such as WSNs [1–4]. Therefore, an accurate energy model is necessary in a very early design stage, before prototyping, to help the efficient design of the physical layer. Moreover, this model should also consider dynamically adjusted packet reception and transmission rates, depending on available energy, to simulate energy-neutral operation (ENO).

Energy harvesting circuits include hardware components for which modeling requires I–V (Current and Voltage) tracking to be reliable. However, as far as we know, the most recent implementations of the classic energy approach in the field lack numerical models of components with an energy level-dependent behavior. As an example, a resistor-capacitor (RC) network presents a different behavior at different energy levels. To address this problem, this paper presents a mathematical I–V model for the design of EH-enabled WSN. The model is called SIVEH (Simulator I–V for EH) and brings about a flexible solution for simulating most EH current circuits.

Moreover, as solar radiation values depend on the final WSN location around the world and along the season, SIVEH implementation allows the use of long solar radiation vectors ( $\frac{J}{m^{2}}$) that can be directly extracted from public databases. Although the prediction of future solar radiation values is out of the scope of this work, the use of registered values provides very realistic information about the variation of the buffered energy during hours, weeks, months or years, according to sunlight progression in a specific world location.

The SIVEH model increases precision compared with the energy approaches used in the literature. Moreover, as SPICE is assumed to be a reference in electronics simulation, SIVEH computational implementation has also been compared with it. SIVEH has shown a maximum derivate error of less than 1% with a significant improvement on simulation speed. The aim is to propose a new model of energy harvesting nodes for wireless sensor network simulation with the following features: high precision, high simulation speed and allowing for complex networks simulation. As a case study, the implementation of an algorithm to reach energy neutral operation (ENO) conditions is proposed.

This paper is organized as follows. Section 2 gives a general related literature overview. Section 3 presents the mathematical approach of SIVEH. Section 4 presents the SIVEH framework with a generic description of energy harvesting circuits and an example describing the implementation of a prototype. Section 5 evaluates SIVEH error and simulation time vs. both an energy approach and SPICE. Section 6 describes the implementation of the SIVEH model. Section 7 carries out a verification by comparing SIVEH outputs with a prototype. Section 8 presents a case study: The ENO condition in WSN nodes. Finally, Section 9 concludes this paper.

## 2. Motivation

Techniques to harvest energy are used to convert energy from ambient sources into electrical energy stored in buffers [5,6], which are usually supercapacitors or rechargeable batteries [7]. Some of the literature has focused on sunlight energy-harvesting circuits, although this research does not usually address networking aspects. Some authors include rechargeable batteries as the main buffer [8,9]. However, batteries have a finite number of charge-discharge cycles, meaning that the use of another solution based on supercapacitors is advisable [9–12]. Moreover, an EH circuit can include both solutions—supercapacitors and backup battery [13,14]. Solar EH circuits currently already incorporate Maximum Power Point Tracking (MPPT) [15,16] and power management hardware features [11].

The energy lifetime of self-powered wireless communication devices depends not only on energy-harvesting capabilities, but also on energy demands, which are rarely static and change as a function of radio operating modes.

The main motivation to work on a new EH model is the need to accurately describe some energy-dependent effects frequently observed in these circuits. I–V tracking is demonstrated to be accurate when components present different power dissipation at either different operating voltages or drawn currents. Previous energy-based approaches calculate capacitor available energy by adding and subtracting harvested and consumed energy, respectively [11,17–19]. However, there are some objections to these works. In [17], energy management components, such as DC-DC converters or low drop-out regulators (LDO) are not considered, so the model is not representative in ultra low power modes. In [19], a constant energy buffer charging efficiency and a constant leakage power are considered with some boundaries. However, as [18] demonstrates, these parameters are not constant, but depend on instant voltage and current conditions. In [18], DC-DC circuits are included, and I–V operating conditions are concluded to be needed. However, the mathematical formulation is very complex and will require many computational resources, which can be efficiently reduced, as this paper shows. Empirical methods and prototyping are used in [11]. However, empirical models cannot always be generalized.

In conclusion, a reliable numerical energy model implies I–V tracking. Nevertheless, in this paper, an alternative is presented with the aim of reducing computational load. The model is called SIVEH and is I–V tracking-based. Computational complexity is reduced, and simulation time is significantly improved using both variables.

Energy management policies have already been studied for networks with energy-harvesting sensor nodes [19–22], *etc.* Power management systems contribute to adjust sensor node consumption to available energy. Thus, SIVEH has also been designed to enable the simulation of energy-neutral operation (ENO) conditions.

However, numerical computing is limited in sensor network simulation, and event-based computer network simulation tools are powerful [22]. This motivated an early implementation of SIVEH in ns3 [23]. This paper addresses the full description of SIVEH for a more general model, which can be implemented in both network simulation tools and numerical computation tools.

## 3. Mathematical Approach

I–V tracking is strongly necessary for an accurate circuit simulation. The SPICE simulator [24], which is based on this principle, is widely used and has been used as a reference in previous EH systems design [8] and energy estimation tools [25]. The objective of SIVEH is to reduce computational load, increasing simulation speed and decreasing memory requirements, but still with high accuracy. To understand the model, basics on EH circuits based on supercapacitors are described in this section.

### 3.1. Supercapacitor Modeling

Supercapacitors are non-ideal rechargeable buffers. Energy stored in them is given by expression (1), where *V_scap_* is the voltage in the capacitor and C is the rated capacitance. Therefore, a supercapacitor voltage decreases, as long as its remaining energy is depleted.

(1) $$E_{\text{scap}}(t)=\frac{1}{2}C{\left(V_{\text{scap}}(t)\right)}^{2}$$

An energy model estimates remaining energy *E_scap_* at time (t + 1) using expression (2). The energy either consumed or harvested at time (t) depends on every component in the system. ΔE calculation increases complexity, which can be reduced with a I–V formulation without accuracy loss.

(2) $$E_{\text{scap}}(t+\Delta t)=E_{\text{scap}}(t)-\Delta E_{\text{consumed}}(t)+\Delta E_{\text{harvested}}(t)$$

Alternatively, capacitor voltage at time (t) is calculated in expression (3). *V_scap_* is directly dependent on current flowing through capacitor (*I_scap_*).

(3) $$V_{\text{scap}}(t)=\frac{1}{C}\int I_{\text{scap}}(t)\text{d}t$$

*I_scap_* is the supercapacitor current at a given time instant (t) and it is calculated as the difference between demands (*I_consumed_*) and harvested current (*I_harvested_*) in expression (4).

(4) $$I_{\text{scap}}(t)=I_{\text{consumed}}(t)-I_{\text{harvested}}(t)$$

Circuit currents are estimated according to the circuit description and expressed in Equation (5), where circuit components have been divided into four groups discussed in Section 3.2.

(5) $$I_{\text{consumed}}(t)=\sum I_{R}(t)+\sum I_{\text{sink}}(t)+\sum I_{DC-DC}(t)+\sum I_{\text{linear}}(t)$$

The presented approach assumes a constant current during each time slot (Δt). Thus, a key issue is the definition of the time slot. This assumption introduces an error to be bounded below an acceptable threshold. As this threshold can be set, as will be later demonstrated, SIVEH uses expression (6) instead of Equation (3) to calculate supercapacitor remaining voltage at the next time slot (Δt).

(6) $$V_{\text{scap}}(t+\Delta t)≃V_{\text{scap}}(t)-\frac{1}{C}I_{\text{scap}}(t)\Delta t$$

### 3.2. Circuit Components

**Figure 1.**
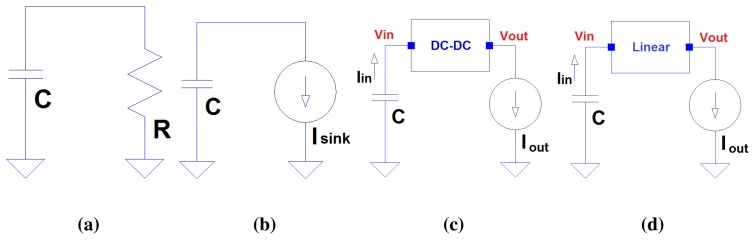
Energy harvesting circuit basic blocks: (a) resistor, (b) current sink, (c) DC-DC converter and (d) linear regulator.

Current demand, *I_consumed_*, is the addition of current drained in every component in the node. Reviewing the most recent EH circuits and the overall sensor node architectures, hardware usually consists of components of the following nature (Figure 1): (1a) resistors; (1b) current sinks; (1c) DC-DC energy management blocks; and (1d) Linear regulators.

#### 3.2.1. Resistors

An examples of resistor abstract-modeled components are resistor network voltage dividers (e.g., feedback networks and comparator threshold). Current leakage in the supercapacitors can be also modeled as an ideal resistor [26].

Energy modeling needs to approximate resistor energy consumption, expression (7), since resistor applied voltage decreases when R is connected to a capacitor, as shown in Figure (1a).

(7) $$\Delta E_{R}(t,\Delta t)≃P_{R}(t)\Delta t=\frac{V_{R}^{2}(t)}{R}\Delta t$$

SIVEH model reduces complexity using expression (8) by Ohm's law.

(8) $$I_{R}(t)=\frac{V_{R}(t)}{R}$$

#### 3.2.2. Current Sinks

Integrated circuits directly connected to supercapacitors dissipate some current, even inactive (quiescent) current. Moreover, components, such as the control unit (MCU), attached sensors or a wireless RF circuit are also modeled as a current basic type described in Figure (1b).

Consumed energy, assuming a constant voltage condition, is expressed in Equation (9).

(9) $$\Delta E_{\text{sink}}(t)≃V_{\text{sink}}(t)I_{\text{sink}}(t)\Delta t$$

As the SIVEH model is formulated in terms of I–V, dissipated currents in expression (10) are directly obtained from vendor data-sheets.

(10) $$I_{\text{sink}}(t)=f(\text{Datasheet})$$

#### 3.2.3. Voltage Regulators

Voltage regulators are frequently observed in harvesting circuits. These electronic components can be divided into two main separate groups: DC-DC converters (Figure 1c) and linear devices (Figure 1d).

##### DC-DC converters

Energy consumed by a DC-DC converter depends on the converter efficiency; expression (11), where *E_out_* is the energy consumed by every circuit element attached to the DC-DC output.

(11) $$\Delta E_{DC-DC}(t)=\frac{E_{\text{out}}(t)}{eff_{DC-DC}}$$

As a I–V formulation is used in SIVEH, input current to DC-DC converters is described using expression (12):

- *V_in_* denotes the voltage measured in the converter input. This case corresponds to the energy buffer voltage.
- *V_out_* denotes the back module supply voltage. This value is fixed beforehand, according to node demands (MCU, sensors, etc.).
- *I_out_* denotes the current dissipated by the modules connected to the converter output.
- *ef f_DC-DC_*: DC-DC converter efficiency is voltage and current operating conditions -dependent [18].

(12) $$I_{in}(t)=\frac{V_{\text{out}}(t)}{V_{in}(t)}\frac{I_{\text{out}}(t)}{eff_{DC-DC}}$$

##### Linear devices

On the other hand, linear regulators and LDO voltage regulators can be considered linear devices (Figure 1d). Exceptionally, diodes can also be considered a part of this group under especial circumstances (e.g., supercapacitor charge using a diode, as in [14]).

Energy consumed using such devices is calculated in expression (13).

(13) $$\Delta E_{\text{linear}}(t)≃V_{in}(t)I_{\text{out}}(t)\Delta t$$

In the SIVEH approach, these elements are ideally modeled by setting input current (*I_in_*) equal to the devices connected to its output current consumption (*I_out_*), as expressed in Equation (14)

(14) $$I_{\text{linear}}(t)=I_{\text{out}}(t)$$

### 3.3. Energy Harvesters

In case of solar EH, supercapacitor recharge depends on solar radiation conditions, solar cells and the hardware between supercapacitors and solar cells. The SIVEH model contemplates these three items to calculate supercapacitor supplied current.

Firstly, solar energy values can be directly imported from solar radiation databases. This off-line process obtains more realistic scenarios with respect to world location conditions and meteorological variability. For example, some national or state local weather services record hourly-integrated solar energy $\frac{J}{m^{2}}$ levels that can be downloaded into comma separated values (.csv) format. $\frac{J}{m^{2}}$ vectors contain enough data to simulate from some hours to several years.

Secondly, solar power average and cell harvested currents are calculated by expressions (16), the ratio between hourly-integrated energy and average power, and (15), cell supplied power mean, respectively. *P_cell_* limit in expression (15) is *P_cellmax_*.

(15) $${\bar{P}}_{\text{cell}}(t)=P_{{\text{cell}}_{\max}}\frac{{\bar{P}}_{\text{solar}}(t)(\frac{W}{m^{2}})}{1000(\frac{W}{m^{2}})}$$

(16) $${\bar{P}}_{\text{solar}}(t)=\frac{{\bar{E}}_{\text{solar}(\text{hourly})}(t)(\frac{J}{m^{2}})}{3600(s)}$$

Thirdly, hardware between supercapacitors and solar cells is required to manage the recharge process and avoid capacitor damage caused by inappropriate operating conditions. Hardware uses include DC-DC converters or linear components. Thus, input power (or energy, since *E* = *P*Δ*t*) recharges the capacitor by expression (12) or (14).

*I_c_ell* is calculated following expression (17) and is limited to 300 mA.

(17) $$I_{\text{cell}}(t)=\frac{{\bar{P}}_{\text{cell}}(t)}{V_{\text{scap}}(t)}\frac{1}{eff_{\text{Block}A}}$$

## 4. Generic EH Circuits Description

**Figure 2.**
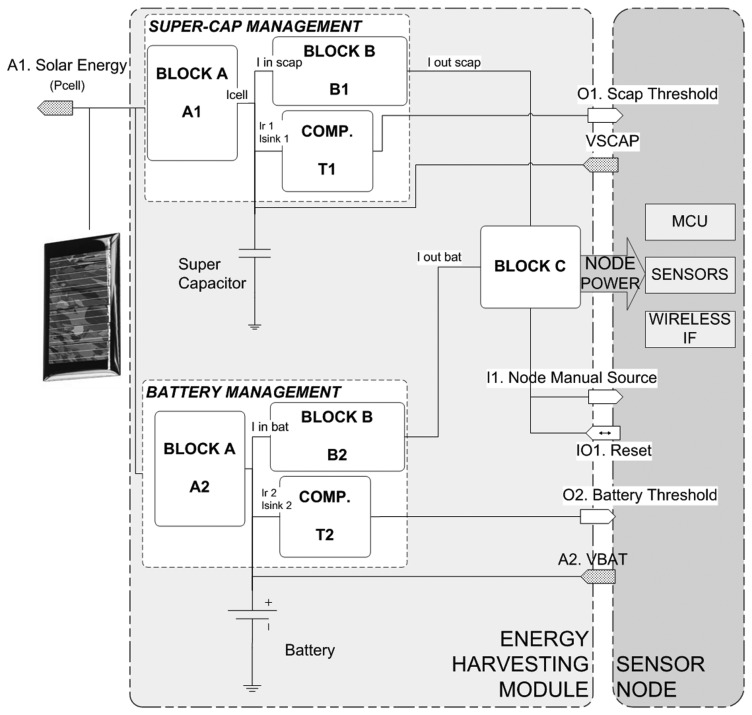
Generic energy harvesting circuit simplified block diagram.

Figure 2 shows a generic EH circuit block diagram. The figure tries to summarize all the recent advances in energy harvesters in a general architecture, embedding the most significant blocks. Additionally, each block is linked with the corresponding model described in Section 3.

The circuit includes two energy buffers. Supercapacitors are preferred as a primary energy buffer. They can be recharged several times very fast and without significant degradation. Since supercapacitor energy is limited and self-discharge is quite high, a secondary buffer is usually included. Both rechargeable and non-rechargeable batteries have been found in the literature.

Since the two types of energy buffers present different specifications, independent A, B and comparer (COMP) blocks have been considered in the design.

- Block A handles buffer recharge using the energy harvested. Although a solar cell is shown in Figure 2, wind and even vibrations can be used as the primary energy source to recharge energy buffers. This block is usually implemented by *DC-DC converters* or *linear devices. Resistors* can also be considered in closed-loop feedback networks. The *current sink* model describes leakage currents.
- Block B allows matching energy buffer output voltage and sensor node operating voltage. This block can also be implemented by means of DC-DC converters and/or linear devices. As in the previous block, some quiescent current is drawn that can be modeled using the current sink model.
- An energy threshold comparer (COMP) is useful for estimating buffered remaining energy and handling flags in the sensor node. This block dissipates some current, modeled as the current sink, and a resistor network, modeled as a resistor.

Block C is a mechanism designed for switching the input supply line to the sensor node between the primary and the secondary energy buffers without MCU piggybacking. It guarantees sensor node stable supply voltage and optimizes the use of energy, optimizing energy consumed from the secondary buffer as long as there is remaining energy in the primary buffer. This block is usually modeled as a linear device with an associated quiescent current, modeled as a current sink.

### 4.1. EH Prototype for Evaluation Purpose Description

As final implementation of the different blocks depends on the actual hardware, an example is proposed: an improved version of [23]. The main innovations are MPPT and a backup battery. Energy storage is mainly carried out by supercapacitors. Two-point-three-volt Panasonic supercapacitors and a Li-Ion non-rechargeable CGR18650DA battery have been included as the main and secondary buffers, respectively. In future work, rechargeable batteries will also be considered. However, for verification purposes, the Li-Ion battery model is available [27] and has been added to SIVEH.

The energy-harvesting circuit is designed with solar cells rated at 4 V open-circuit and 3.5 peak voltage, 48.5 mA short-circuit and 45 mA peak current. The circuit connects either one cell or several, depending on energy demands. Block A1 in Figure 2 collects energy from solar cells and stores it in two 2.3 V supercapacitors connected in series. This block incorporates a Texas Instruments (TI) BQ25504 integrated circuit [28] that is a PWM converter to implement the MPPT algorithm for harvesting energy from low input sources (80 mV) with an efficiency above 80%.

The behavior of BQ25504 is as follows:

- If supercapacitor voltage is below 1.8 V, a cold start procedure runs, and supercapacitor recharge current (*I_cell_*) is calculated using Equation (18) according to [23].
  (18) $$I_{\text{cell}}(t)=I_{\text{cell}\max}\frac{{\bar{P}}_{\text{solar}}(t)(\frac{W}{m^{2}})}{1000(\frac{W}{m^{2}})}$$
- In another case, the MPPT algorithm is carried out, and *I_cell_* is calculated using expression (19, a variation of the proposed current equation for DC-DC converters (12).
  (19) $$I_{\text{cell}}(t)=\frac{{\bar{P}}_{\text{cell}}(t)}{V_{\text{scap}}(t)eff_{DC-DC}}$$
- In any case, current is limited to 300 mA.

Since supercapacitor voltage decreases when stored energy is drained, block B1 provides a stable 3.3 V MCU supply voltage, while supercapacitor voltage is between 1 V and 4.6 V. This block includes, for testing every basic circuit described in Figure 1, a DC-DC step-up converter (L6290 ST Microelectronics [29]) together with a TI TPS782333 LDO [28]. Block B behavior is modeled as follows:

- If supercapacitor voltage is below 1 V, block B does not work properly, and only quiescent current (the current sink model) is dissipated according to datasheet values.
- If supercapacitor voltage is between 1 V and 3.3 V, block B acts as a 3.3 V step-up converter. The DC-DC converter model is used (expression (12)), adding the correspondent quiescent current (the current sink model).
- If supercapacitor voltage is above 3.3 V, block B acts as a linear regulator. The linear device model (expression (14)) is used in this case.

A flag is set when supercapacitor voltage is below 1 V. The COM block consists of a voltage comparator that drains 400 nA (the current sink model) and a 1 *M*Ω voltage divider resistor network (resistor model).

Since a 3.6 V non-rechargeable battery has been included into the prototype, blocks A2 and B2 are disabled. *Iin_bat_* and *Iout_bat_* are equal in Figure 2. Both are renamed as *I_bat_*.

Block C switches to backup battery when *V_scap_* is below 1 V (minimum operating voltage of block B1). It guarantees a 3.3 V supply voltage. This block includes a TPS3606-33 integrated circuit (Texas Instruments) with power dissipation below 40 μA (the current sink model).

## 5. Performance Analysis

As already mentioned in Section 3, time slot approximation introduces a numerical error. Errors can be quantified using Maclaurin series and the Taylor error theorem, as shown in the Appendices. Errors are summarized in Table 1. SIVEH error is bounded by defining a simulation time slot (Δt) according to the capacitor. For example, a DC-DC converter, C = 10 mF, eff = 92% and Vout = 2.3 V, attached to a resistor of 100 Ω, shows an error below 1% for any Δt below 3.9 ms.

### 5.1. Simulation Analysis

The use of analog electronic circuit simulators, such as SPICE, is widely accepted. The basic circuits in Figure 1 have been simulated using a SPICE-based simulator (LT-SPICE [30]), the SIVEH model and the energy approach. The objective of this section is to evaluate both the accuracy and simulation performance.

**Figure 3.**
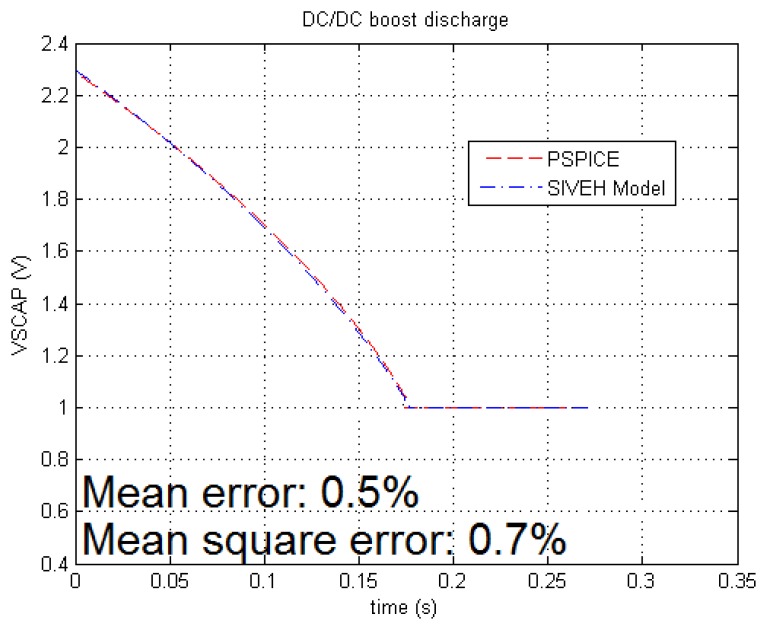
SPICE vs. MatLab step-up DC-DC converter.

DC-DC comparison is carried out using L6920 of ST [29] and outputs plotted in Figure 3 that shows a model deviation from the SPICE reference less than 0.7%. The mean error and mean square error [Me, Mqe] in *I_sink_* and resistors are [0.04%, 0.04%] and [0.008%, 0.012%], respectively. Energy tracking and SIVEH present barely the same accuracy for all cases: 0.1% maximum deviation measured in the resistor case in which both solutions present the some error.

However, SPICE simulation time is very lengthy with DC-DC converters. For example, LT-SPICE takes around 20 h to simulate the complete discharge of a 10 F capacitor. However, numerical computing is almost constant, independent of component complexity. Table 2 shows a practical comparative example. The higher performance speed of the SIVEH approach is shown in both SPICE and the numerical energy approach. For example, simulation speed increases, on average, 240% compared with the energy models.

## 6. SIVEH Model Implementation

**Figure 4.**
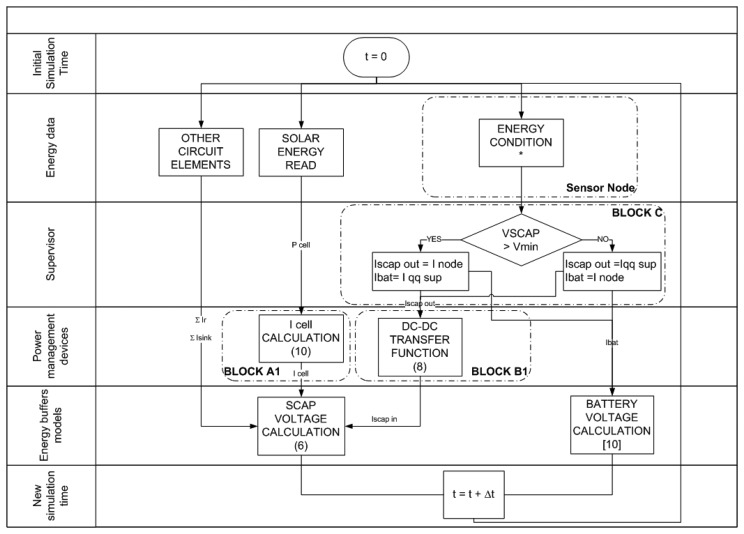
SIVEH flow diagram.

The SIVEH model has been implemented using a numerical computing language for fast evaluation. It has also successfully been implemented in discrete-event network simulators, such as ns-3 [31]. Figure 4 presents the overall abstraction flow diagram of SIVEH.

1. *Energy data* depends on the sensor node operating mode at the current time slot (t) and includes three tasks:
  (a) Solar energy is read from the solar database, and cell power is calculated using expression (15).
  (b) Supercapacitor self-discharge, as well as resistor networks, IC quiescent current, *etc.*, directly attached to the supercapacitors, are calculated using expressions (8) and (10).
  (c) Sensor node current consumption is set according to the current operating mode.
2. *Supervisor*: Block C in Figure 2 checks for a minimum threshold condition in *V_scap_* and determines whether the sensor node will be powered by the supercapacitor (*I_scapout_*) or by the battery (*I_bat_*). Some quiescent current is drawn by the non-connected energy source.
3. *Power management devices*: Voltage regulator demands are calculated to estimate the supercapac-itor drawn current.
  (a) Expressions (12) and (14) are calculated at this flow diagram stage.
  (b) Energy dissipated by DC-DC components themselves is also calculated with expression (10).
  (c) Block A1 calculates *I_cell_*(t).
4. *Energy buffers models*: Buffering voltages are updated.
  (a) Supercapacitor voltage is updated with expressions (6) and (4), as the addition of *I_r_*, *I_sink_*, *I_cell_* and *I_scapin_*, shown in Figure 4.
  (b) Battery voltage (*V_bat_*) is updated using the model proposed in [27].
5. *New simulation time*: At the end of the flow diagram, time is also updated to the next period (t + 1).

## 7. Model Verification

SIVEH model estimations have been compared with the prototype. For these experiments, the sensor node was programmed with the state diagram in Figure 7: sleep time: 7 s and 10 μW; duty cycle 7.6%: Rx reception 512 ms and 24 mW; Tx transmission: 64 ms and 108 mW. Consumption values have been collected from [23,32].

**Figure 5.**
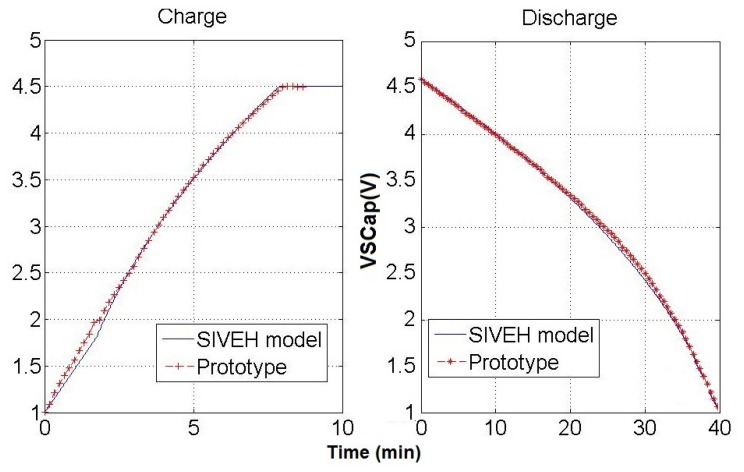
SIVEH outputs compared with real prototype energy stored; charge and discharge cycles. C = 5F, Δt = 1 s.

Two experiments have been carried out. Firstly, indoor experiments to force fast recharge and discharge (860 $\frac{W}{m^{2}}$ were measured in recharge and 0 W/m2 in discharge). Figure 5 shows the indoor experimental results. Both SIVEH and prototype outputs match with a slide deviation of 2%.

**Figure 6.**
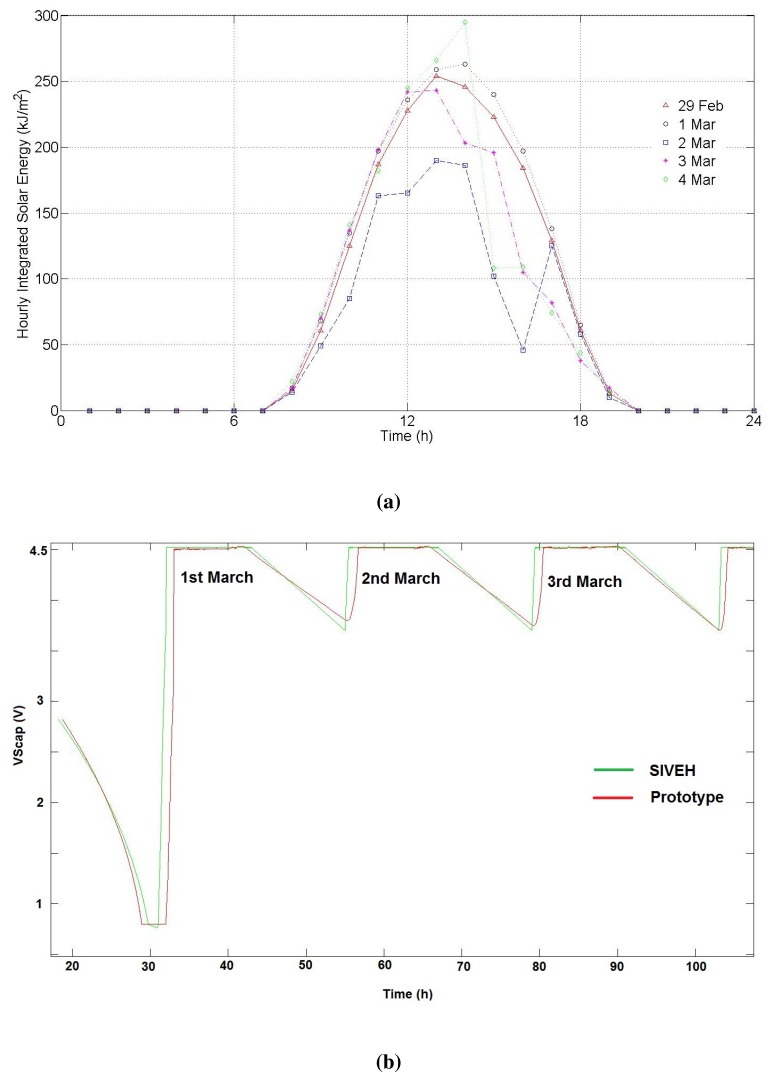
Four-day validation experiment. (**a**) Solar conditions from February 29 to March 1, 2012; (**b**) SIVEH predictive results vs. prototype outcomes. Four-day outdoor comparison. C = 5F Δt = 1 s.

Secondly, the prototype was located outdoors from March 1 to 4. The experiment was carried out in a river 7.8 km away from a AEMet weather station placed at 39° 29′ 7” N–0° 28′ 28”. The AEMet solar record is shown in Figure 6a.

The experiment started on February 29, 2012, at 8 pm. The initial *V_scap_* was 2.8 V. Discharge reached 1 V before sunrise (see Figure 6b), leaving the node without enough energy to communicate. Both simulated and real curves show the same effect. However, the simulation curve starts the supercapacitor recharge before the prototype. This effect is given by the one-hour time slot grain of the database. While the value observed by the prototype varies continuously, the simulation uses a sample hourly-integrated solar energy ( $\frac{J}{m^{2}}$) level, which means an error experimentally observed at sunrise and sunset of 0.1 V in the worst case; Figure 6b. From sunrise to sunset, both lines match. Figure 6a shows solar conditions during the experiment.

The low consumption of the modem architecture and the energy harvesting circuit, as well as the quick recharge of supercapacitors at sunrise make it possible to maintain high voltage levels in the supercapacitor during the whole day, even in cloudy weather (see March 2 in Figure 6a). It should be noted that all simulation average values, obtained from manufacturer datasheets, have been used for all components. Statistically distributed results can also be obtained, introducing randomness in the nominal values of components, which can also be obtained in the same datasheets. Experiments should then be repeated as needed.

## 8. Case Study: Energy Neutral Operation

Energy neutral operation (ENO) is the use of harvested energy at an appropriate rate, such that the system continues to operate perennially [19]. The definition of energy neutral operation needs to be evaluated from a very early stage of the design. This implies simultaneous cross layer work on physical design decisions and management power policies.

**Figure 7.**
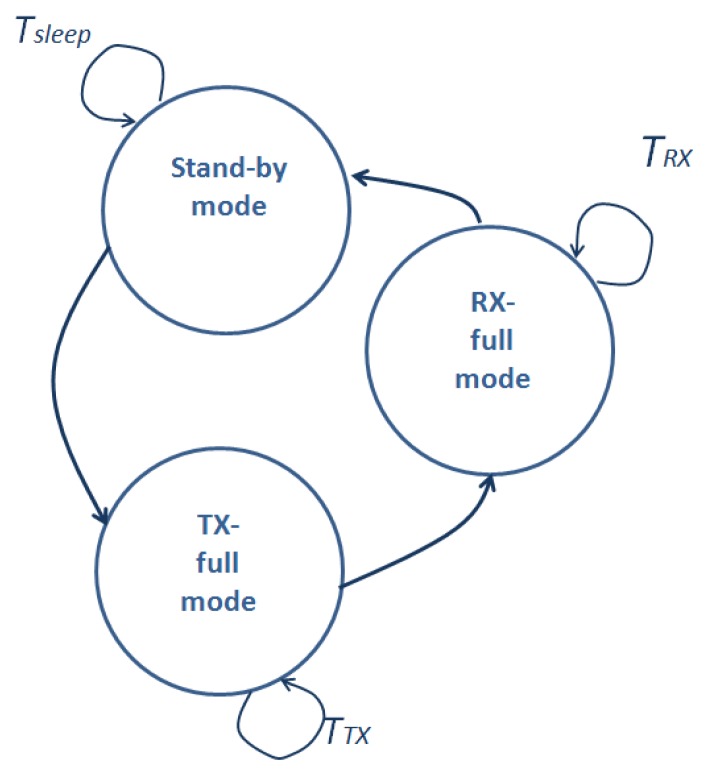
Sensor node state basic diagram.

This section shows an example of the ENO condition in a system with the basic state diagram shown in Figure 7, which will be dynamically re-adjusted (*T_sleep_*, *T_RX_* or *T_TX_*).

Following the state diagram in Figure 7, node average current consumption is calculated with Equation (20), whose reformulation supports Figure 7 extensions.

(20) $${\bar{I}}_{\text{node}}=\frac{I_{TX}T_{TX}+I_{RX}T_{RX}+I_{\text{sleep}}T_{\text{sleep}}}{T_{TX}+T_{RX}+T_{\text{sleep}}}$$

ENO is based on the law of conservation of energy: harvested energy must be equal or greater (excess would be stored) than dissipated energy; Equation (21).

(21) $$\begin{array}{l} E_{\text{harvested}}\geq E_{\text{dissipated}}\\ P_{\text{harvested}}\geq P_{\text{dissipated}} \end{array}$$

Assuming *V_scap_* is higher than a minimum threshold, *P_harvested_* is given in expression (22). This threshold is 1.8 V and assures that the BQ25504 energy harvester operates with a MPPT algorithm.

(22) $$P_{\text{harvested}}=P_{\text{cell}}\cdot eff_{\text{BLOCK}A1}=P_{\text{cell}\max}\frac{{\bar{P}}_{\text{solar}}(t)(\frac{W}{m^{2}})}{1,000(\frac{W}{m^{2}})}eff_{\text{BLOCK}A1}$$

*P_dissipated_* depends on sensor node power consumption requirements (*P_nodeef f_*, since Block B1 efficiency has to be considered), quiescent and leakage currents (*I_sinkeq_*) and the circuit resistor equivalent value (*R_eq_*)*. P_nodeeff_* is usually the most energy-demanding element. Therefore, *P_dissipated_* can be approximated with expression (20). Thus, the initial ENO premise (21) is expanded in (23).

(23) $$P_{\text{cell}\max}\frac{{\bar{P}}_{\text{solar}}(t)(\frac{W}{m^{2}})}{1,000(\frac{W}{m^{2}})}eff_{\text{BLOCK}A1}\geq \frac{V_{\text{node}}}{eff_{\text{BLOCK}B1}}\frac{I_{TX}T_{TX}+I_{RX}T_{RX}+I_{\text{sleep}}T_{\text{sleep}}}{T_{TX}+T_{RX}+T_{\text{sleep}}}$$

Node sleep time is calculated from Equation (23) and is dynamically adjusted using the algorithm, defined below as the ENO Algorithm. Sleep time variation increases or decreases the packet transmission rate in order to consume energy proportionally to the harvested energy. Additionally, the ENO Algorithm sets the energy safe maximum boundary (*V_scap_* = 4.5 V) and minimum and maximum sleep times.

**Listing 1.** Node sleep time basic decision algorithm using ENO**int** ENO_algorithm (**int** Vscap, **int** Pcell, **int** Tsleep max, **int** Tsleep min)**{****if** (Vscap>4.5 V & Pcell >=0.2*Pcell_max) Tsleep = (eq 22);**else** Tsleep = Tsleep / rho;**if** (Tsleep > Tsleep_max) Tsleep=Tsleep max;**else** **if** (Tsleep < Tsleep_min)  Tsleep=Tsleep min;**return** (Tsleep);}

The following simulations analyze the use of ENO condition. Simulations were set on December 8. The ENO settings were: *T_s_*_leep_*_min_* = 0 s and *_Tsl_*_eepm_*_ax_* = 40 s.

**Figure 8.**
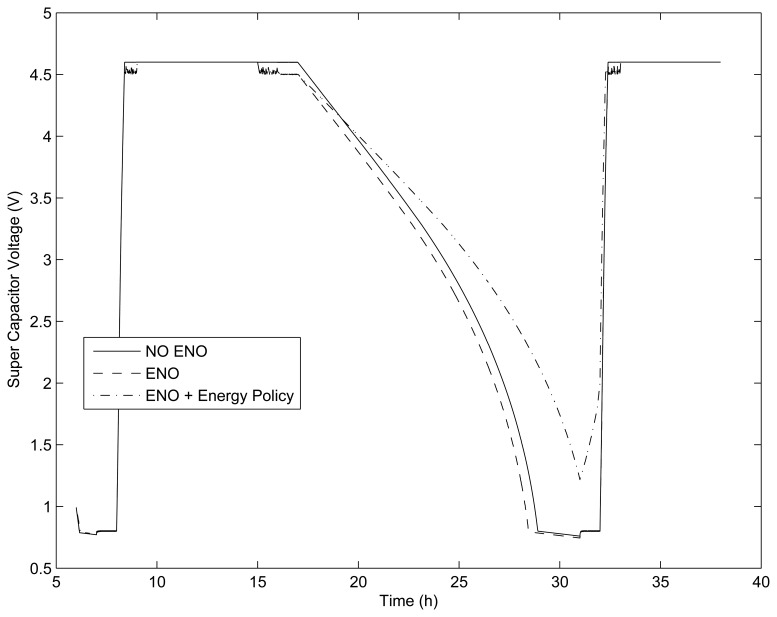
Comparison of energy-neutral operation (ENO) and NO ENO policies.

The evolution of the energy in the supercapacitors is shown in Figure 8.

- Simulation without the ENO condition: 2,614 cycles (1.36 cycles per minute). The output showed that the node needed to be powered by the backup battery for 3 h.
- Simulation with ENO condition: 67,942 cycles (35.3 cycles per minute). The output showed that the node needed to be powered by the backup battery for 3.5 h.

In the SIVEH numerical model, it is possible to introduce power management policies based on probabilities. For instance, sleep time can be conditioned to an external variable, such as *I_ce_*_ll_. When *I_cell_* decreases below 20% of *I_cell_* maximum value, the packet transmission rate decreases into (1 — *ρ*), for example, with a *ρ* = 10%:

- Simulation with ENO condition + *ρ* 10%: 68,571 received messages and 67,419 transmitted messages (35.7 cycles per minute in rechargeable conditions and 35.1 cycles per minute at night). With this adjustment, no battery energy was required.

These results show that a static duty cycle is not really efficient. The ENO condition enables a dynamic adjustment of transmission and reception packet rates for harvested energy which becomes more profitable when taking into account solar radiation differences observed between summer and winter in many world locations.

## 9. Conclusions

This paper presents a numerical model to simulate capacitor energy storage with solar energy-harvesting applied to WSN nodes (SIVEH). The mathematical basis and error boundaries have been accurately described. The proposed model is computationally faster than both an energy-based formulation and a SPICE model. For example, SIVEH reaches a simulation speed up to 54,678 times faster than a SPICE simulation of DC-DC components. Moreover, SIVEH presents an accuracy of 98% of a SPICE reference model.

The model has been designed to support any state-of-the-art energy harvesting platforms, after an extensive analysis, and verified by comparing simulations with a real prototype with less than 2% maximum experimental error.

The model versatility allows for introducing additional features, such as the ENO condition. An example demonstrates that an efficient use of harvested energy could increase communication cycle rates up to 25.9% on average.

Beyond that, the model is enhanced with the ability to work with ( $\frac{J}{m^{2}}$) solar radiation vectors. Thus, the model is a tool that enables an efficient design of the hardware architecture, according to the power management policies specified for the system and the specific world location of the final system.

As a summary, the model is generic and wide enough to be able to simulate any energy harvesting circuit composed of the typical elements of these circuits.

Thus, with the accuracy observed in SIVEH, many architectural design decisions and predictions can be analyzed without requiring a prototype.

## Figures and Tables

**Table 1.** Simulator I-V for EH (SIVEH) and energy models of the basic circuit elements.

		**SIVEH**	**Energy**
Resistor	Proposed	$\frac{V_{R}(t)}{R}$	$\frac{V_{R}^{2}(t)}{R}\Delta t$
	Error	$v_{c}(t)\frac{{\left(\frac{\Delta t}{RC}\right)}^{2}}{2!}$	$\frac{v_{c}{\left(t\right)}^{2}\Delta t^{2}}{R^{2}C}$
Current Sink	Proposed	*f*(*V*,…)	*V_sink_*(*t*) *I_sink_*(*t*)Δt
	Error	0	$\frac{I_{\text{scap}}{\left(t\right)}^{2}\Delta t^{2}}{2C}$
DC-DC Converter	Proposed	$\frac{V_{out}(t)}{V_{in}(t)}\frac{I_{out}(t)}{eff_{DC-DC}}$	$\frac{E_{out}(t)}{eff_{DC-DC}}$
	Error	$\frac{1}{2v_{c}}{\left(\frac{I_{out}V_{out}\Delta t}{Ceff_{DC-DC}}\right)}^{2}$	0
Linear Devices	Proposed	I*_linear_*(t)=I*_out_*(t)	*V_in_*(*t*)*I_out_*(*t*)*Δt*
	Error	0	$\frac{I_{out}{\left(t\right)}^{2}\Delta t^{2}}{2C}$

**Table 2.** Execution time (in seconds) of SPICE, the numerical energy model and SIVEH to simulate a supercapacitor complete discharge process (initial conditions: R = 100 Ω; *I_sink_* = 10 mA; Δt = 1 ms; C = 10 mF; *V_scap_*(t = 0) = 2.3 V; L6920-DB DC-DC); LT3020 linear regulator.

**Case**	**(I) SPICE**	**(II) Energy**	**(III) SIVEH**
Resistor	0.68	0.7966	0.2077
Current sink	0.30	0.111	0.0495
DC-DC	721.76	0.0132	0.0132
Linear	0.87	0.059	0.0287

## A. Appendices

### A.1. SIVEH Model Error Calculation

The appendix analyzes error derivated from resistors and DC-DC devices modeling.

#### 1.1.1. Resistors

Differential Equation (24) describes the behavior of the basic RC circuit shown in Figure (1a). The exact solution for a certain time slot (t) is shown in Equation (25).

(24) $$0=\frac{1}{C}\int i(t)\text{d}t+Ri(t)$$

(25) $$v_{c}(t+\Delta t)=v_{c}(t)e^{\frac{-\Delta t}{RC}}$$

This expression can be approximated by a finite number of terms using Taylor series. In fact, since Δ t is very small (the Δ t≪RC condition must be fulfilled), the Maclaurin series is centered on zero (expression (26)).

(26) $$v_{c}(t+\Delta t)=v_{c}(t)e^{\frac{-\Delta t}{RC}}≃v_{c}(t)\sum_{n=0}^{\infty }\frac{{\left(\frac{-\Delta t}{RC}\right)}^{n}}{n!}=v_{c}(t)(1-\frac{\frac{\Delta t}{RC}}{1}+\frac{\left(\frac{\Delta t}{RC}\right)}{2!}-\ldots )$$

Equation (26) can be rewritten, including SIVEH approximation, plus some additional error (27). Error is bounded using the Taylor theorem and expressed in Equation (28).

(27) $$v_{c}(t+\Delta t)=v_{c}(t)-v_{c}(t)\frac{\Delta t}{RC}+\text{Error}(\Delta t)$$

(28) $$\text{Error}(\Delta t)\leq R_{2}(\Delta t)=v_{c}(t)\frac{{\left(\frac{\Delta t}{RC}\right)}^{2}}{2!}$$

#### 1.1.2. DC-DC Converters

Using a similar procedure, DC-DC converter behavior is described by differential Equation (29), with Equation (30) as an exact solution and with Maclaurin series in Equation (31).

(29) $$v_{c}(t+\Delta t)=-\frac{1}{C}\int i(t)\text{d}t=-\frac{1}{C}\int I_{\text{out}}(t)\frac{V_{\text{out}}(t)}{v_{c}(t)}\frac{1}{eff_{DC-DC}}\text{d}t$$

(30) $$v_{c}(t+\Delta t)=\sqrt{v_{c}^{2}(t)-\frac{2}{C}I_{\text{out}}(t)\frac{1}{eff_{DC-CD}}V_{o}ut(t)\Delta t}$$

(31) $$v_{c}(t+\Delta t)≃v_{c}(t)\overset{\infty }{\underset{n=0}{\text{∑}}}\frac{{\left(-1\right)}^{n-1}(2n-1)!!}{2^{n}n!}{\left(\frac{-2\cdot I_{\text{out}}(t)}{C\cdot eff_{DC-DC}}\frac{V_{\text{out}}(t)}{v_{c}(t)}\Delta t\right)}^{n}$$

Expression (31) includes SIVEH approximation, and the boundary is shown in Equation (32). Taylor's algorithm calculates this error in expression (33).

(32) $$v_{c}(t+\Delta t)=v_{c}(t)-\frac{1}{C}\frac{V_{\text{out}}(t)}{v_{c}(t)}\frac{I_{\text{out}}(t)}{eff_{DC-DC}}+\text{Error}(\Delta t)$$

(33) $$\text{Error}(\Delta t)\leq R_{2}(\Delta t)=v_{c}(t)\frac{1}{8}{\left(-\frac{2}{C}I_{\text{out}}(t)\frac{1}{eff_{DC-DC}}\frac{V_{\text{out}}(t)}{v_{c}(t)}\Delta t\right)}^{2}$$

### A.2. Energy Model Error Calculation

Resistors, current sinks and linear device approximation need to be analyzed. Equation (34) calculates energy consumed by every element.

(34) $$\Delta E(\Delta t)=\int_{t}^{t+\Delta t}I_{\text{scap}}(t)v_{c}(t)\text{d}t$$

#### 1.2.1. Resistor

Equation (35) is the differential equation to be solved. Equation (36) is the exact solution and the Maclaurin series approximation.

(35) $$\Delta E(\Delta t)=\int_{t}^{t+\Delta t}v_{c}(t)\frac{v_{c}(t)}{R}\text{d}t$$

(36) $$\Delta E(t+\Delta t)=E(t)(1-e^{\frac{-2\Delta t}{RC}})≃E(t)(1-\sum_{n=0}^{\infty }\frac{{\left(\frac{-2\Delta t}{RC}\right)}^{n}}{n!})=\frac{v_{c}{\left(t\right)}^{2}}{R}\Delta t+\text{Error}(\Delta t)$$

According to Taylor's theorem, the error can be bounded by expression (37).

(37) $$\text{Error}(\Delta t)\leq R_{2}(\Delta t)=\frac{v_{c}{\left(t\right)}^{2}\Delta t^{2}}{R^{2}C}$$

#### 1.2.2. Current Sink

The proposed differential Equation (38) is solved in Equation (39). Error is calculated in expression (40).

(38) $$\Delta E(\Delta t)=\int_{t}^{t+\Delta t}(v_{c}(t)-\frac{I_{\text{scap}}(t)}{C}t)I_{\text{scap}}(t)\text{d}t$$

(39) $$\Delta E(\Delta t)=\int_{t}^{t+\Delta t}\left(v_{c}(t)I_{\text{scap}}(t)\Delta t-\frac{I_{\text{scap}}{\left(t\right)}^{2}\Delta t^{2}}{2C}\right)$$

(40) $$\text{Error}(\Delta t)=\frac{I_{\text{scap}}{\left(t\right)}^{2}\Delta t^{2}}{2C}$$

#### 1.2.3. Linear Energy Management Device

The error of linear devices is calculated in expression (41).

(41) $$\text{Error}(\Delta t)=\frac{I_{\text{out}}{\left(t\right)}^{2}\Delta t^{2}}{2C}$$
